# Effect of the fluorescent probes ThT and ANS on the mature amyloid fibrils

**DOI:** 10.1080/19336896.2020.1720487

**Published:** 2020-02-03

**Authors:** M. I. Sulatsky, A. I. Sulatskaya, O. I. Povarova, Iu. A. Antifeeva, I. M. Kuznetsova, K. K. Turoverov

**Affiliations:** a Laboratory of Cell Morphology, Institute of Cytology Russian Academy of Science, St. Petersburg, Russia; b Laboratory of Structural Dynamics, Stability and Folding of Proteins, Institute of Cytology Russian Academy of Science, St. Petersburg, Russia; c Institute of Physics, Nanotechnology and Telecommunications, Peter the Great St. Petersburg Polytechnic University, St. Petersburg, Russia

**Keywords:** Amyloid fibrils, secondary structure, morphology, fluorescent probes, 1-anilino-8-naphthalene sulfonate (ANS), thioflavin T (ThT), ultrasonication

## Abstract

Fluorescent probes thioflavin T (ThT) and 1-anilino-8-naphthalene sulfonate (ANS) are widely used to study amyloid fibrils that accumulate in the body of patients with serious diseases, such as Alzheimer’s, Parkinson’s, prion diseases, etc. However, the possible effect of these probes on amyloid fibrils is not well understood. In this work, we investigated the photophysical characteristics, structure, and morphology of mature amyloid fibrils formed from two model proteins, insulin and lysozyme, in the presence of ThT and ANS. It turned out that ANS affects the secondary structure of amyloids (shown for fibrils formed from insulin and lysozyme) and their fibers clusterization (valid for lysozyme fibrils), while ThT has no such effects. These results confirm the differences in the mechanisms of these dyes interaction with amyloid fibrils. Observed effect of ANS was explained by the electrostatic interactions between the dye molecule and cationic groups of amyloid-forming proteins (unlike hydrophobic binding of ThT) that induce amyloids conformational changes. This interaction leads to weakening repulsion between positive charges of amyloid fibrils and can promote their clusterization. It was shown that when fibrillogenesis conditions and, consequently, fibrils structure is changing, as well as during defragmentation of amyloids by ultrasonication, the influence of ANS to amyloids does not change, which indicates the universality of the detected effects. Based on the obtained results, it was concluded that ANS should be used cautiously for the study of amyloid fibrils, since this fluorescence probe have a direct effect on the object of study.

## Introduction

The formation of ordered protein aggregates, amyloid fibrils, is associated with serious human diseases, such as Alzheimer’s, Parkinson’s, prion diseases, etc [1–7]. Recent studies show that amyloids can also perform a number of important physiological functions [8–10]. In addition, it was shown that the unique biophysical and physicochemical properties of proteins in the state of amyloid fibrils open prospects for their use in bionanotechnology [11,12]. Thus, the study of the mechanisms of formation, structure and stability of amyloid fibrils is currently a relevant practical task.

A wide range of physicochemical methods is used to study amyloid fibrils [13–15]. One of these approaches is the use of fluorescent probes that change their photophysical characteristics during its interaction with amyloid fibrils. Such probes, in particular, are thioflavin T (ThT) and 1-anilino-8-naphthalene sulfonate (ANS). The benzothiazole dye ThT is the ‘gold standard’ for amyloid investigation [16–21]. This is due to a significant increase in fluorescence intensity and fluorescence lifetime of the dye accompanying its binding to amyloid fibrils in comparison to that of free ThT in aqueous solution which is caused by the molecular rotor nature of this dye [22,23]. In addition, the interaction of the dye with fibrils has a high specificity. ThT does not interact with globular proteins in a native state (other than with acetylcholinesterase [24] and serum albumins [25,26]), with molten globule and unfolded states or amorphous aggregates of proteins. Due to its unique properties, ThT is a sensitive tool for diagnostics of amyloid fibrils formation, studying the kinetics of fibrillogenesis, and more recently for the study their structure [27–31].

The hydrophobic probe ANS is commonly used in protein folding studies as a tool for the detection of the formation of partially folded intermediates with solvent-exposed hydrophobic clusters (see, e.g. [32–35]). This dye has low fluorescence quantum yield in polar environments, such as aqueous solutions, but its fluorescence is dramatically increased in nonpolar environments. An increase in fluorescence intensity and a blue shift in the emission maximum wavelength are generally assumed to reflect dye binding to hydrophobic sites in proteins. In this connection, for a long time, ANS was considered as ‘gold standard’ for the kinetic and equilibrium investigation of protein folding/unfolding and characterization of partially folded intermediates, such as molten globules. Just as a marker of the protein’s intermediate states ANS is used in the examination of amyloid fibrils formation (including to assess the influence of various external factors on this process), as well as there are a number of works in which ANS is also used in the study of mature fibrils [36–39].

The effectiveness of the use of fluorescent probes for the amyloids investigation largely depends on the correctness of ideas about the mechanism of the dyes interaction with fibrils and their influence on the studied objects. There are a number of works showing that ThT can change the rate of protein aggregation and even inhibit the growth of amyloid fibrils [40,41]. At the same time, the question of the ThT and ANS effect on mature amyloid fibrils remains unclear. The solution to this question is essential, in particular, for the correct use of these dyes in studying the polymorphism of amyloid fibrils and the relationship of the amyloids structure with their cytotoxicity. In this work, using a number of spectroscopic approaches, as well as methods for visualizing of amyloid fibrils, we studied ANS and ThT influence on the photophysical properties, secondary structure, and morphology of amyloid fibrils.

## Results

### Amyloid fibrils formed from insulin and lysozyme under the same conditions have differences in the secondary structure and tendency to clasterization

For amyloid fibrils preparation two amyloidogenic proteins, insulin and lysozyme, which have significant differences in the primary, secondary and tertiary structure, were selected. Aggregates of these proteins are widely used model objects for amyloids study. In addition, their accumulation in the body of patients leads to the development of a hereditary systemic lysozyme amyloidosis and insulin-derived amyloidosis [7,42,43]. *In vitro* proteins fibrillogenesis was induced by their incubation in a buffer containing denaturation agent (3 M guanidine hydrochloride) at a temperature of 57°C [44]. After the formation of mature amyloid fibrils, aggregates were transferred to distilled water to exclude the influence of the buffer components on the experimental results. The stability of amyloid fibrils in water solution was experimentally confirmed.

**Figure 1.**
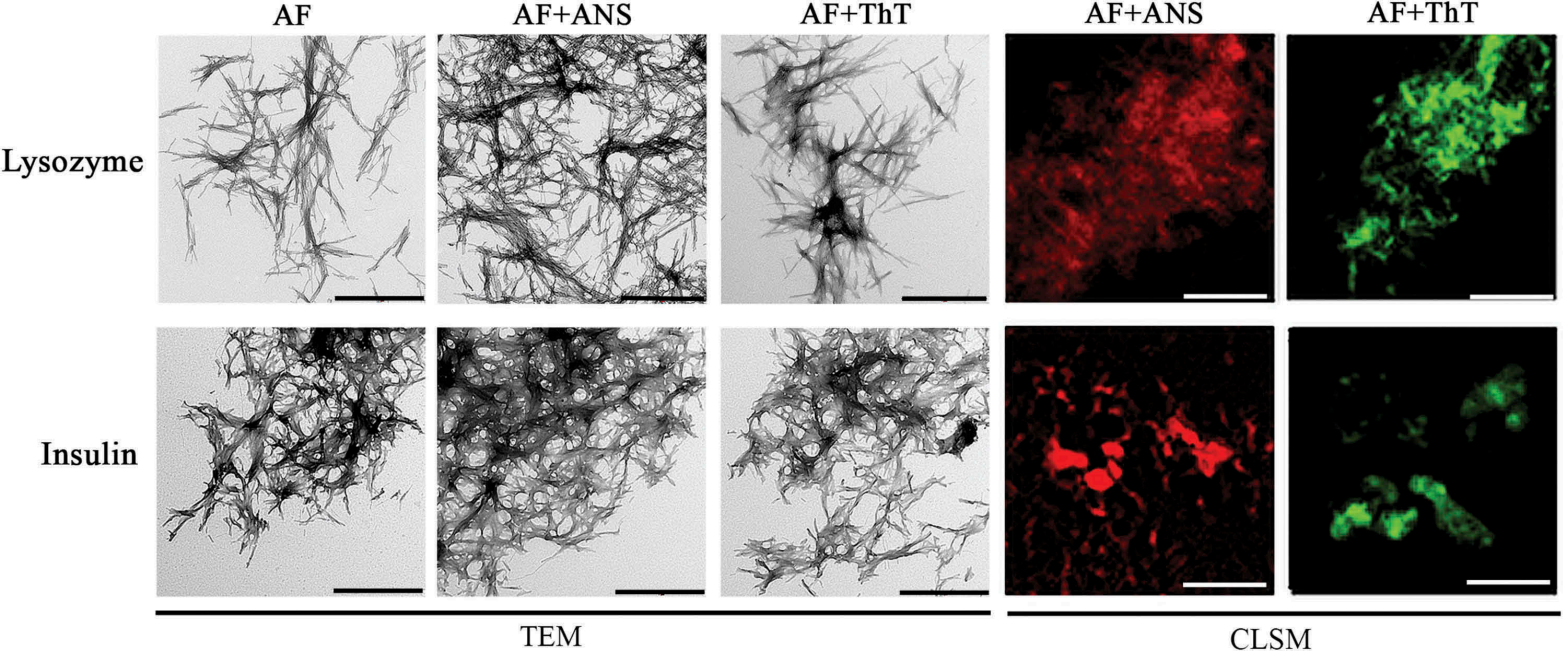
Morphology and staining by fluorescent probes of amyloid fibrils formed from lysozyme (top panels) and insulin (bottom panels) using of buffer solution with pH 6.3. Left panels show transmission electron microscopy (TEM) images of amyloid fibrils in the absence (AF) and in the presence of fluorescent probes ANS (AF+ANS) and ThT (AF+ThT). Data of confocal laser scanning microscopy (CLSM, Right panels) show amyloids stained by these fluorescent probes. The scale bars on the TEM and CLSM images are equal to 1 and 4 µm, respectively.

**Figure 2.**
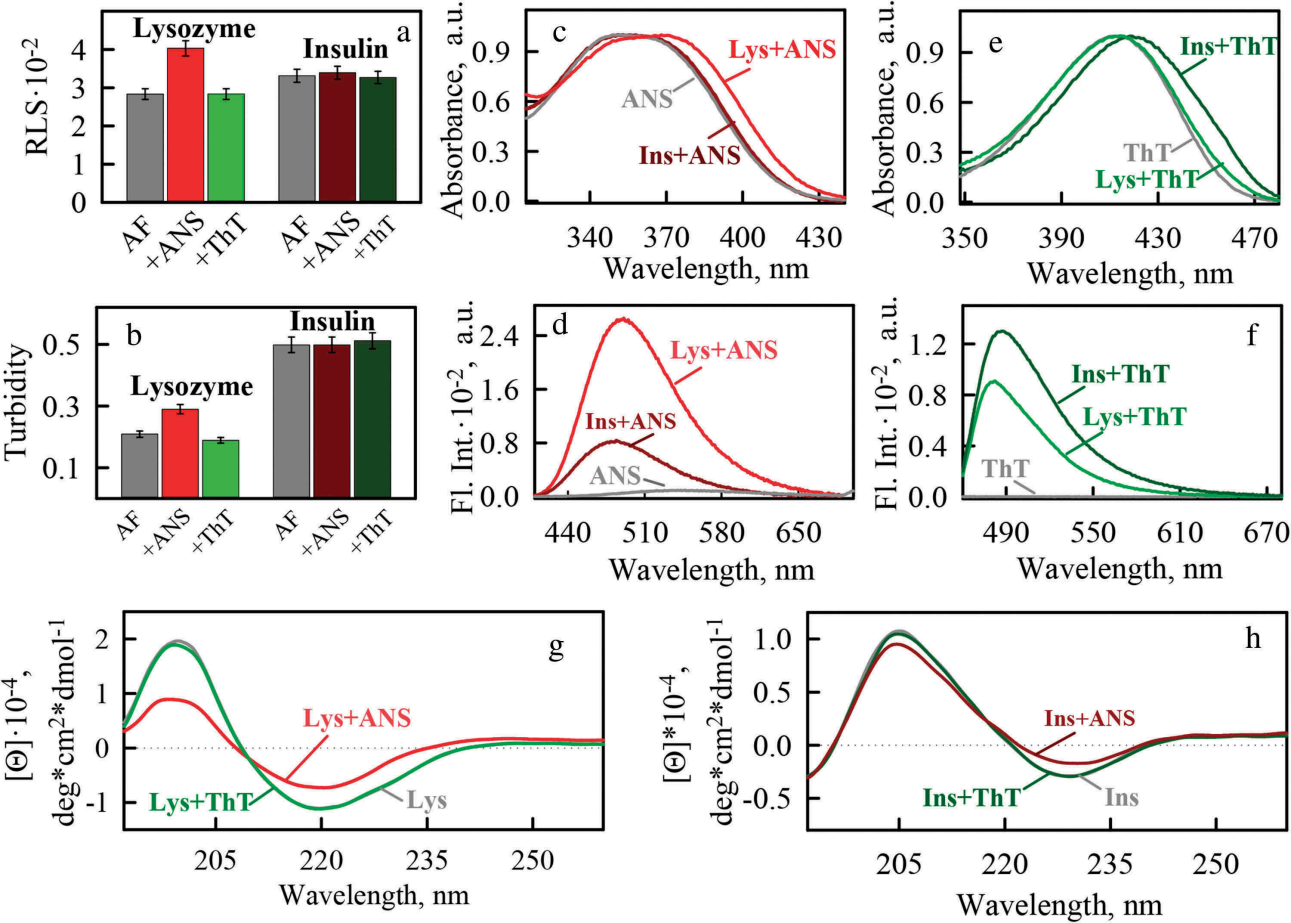
Photophysical properties of amyloid fibrils formed from lysozyme and insulin using of buffer solution with pH 6.3 and their binding to fluorescent probes ThT and ANS. (a) -Rayleigh Light Scattering (RLS) and (b) – turbidity (at λ = 530 nm) of amyloids. (c) – normalized absorbance and (d) – fluorescence spectra of ANS in free (ANS) and bound to lysozyme (Lys+ANS) and insulin (Ins+ANS) amyloid fibrils states. (e) – normalized absorbance and (f) – fluorescence spectra of ThT in free (ThT) and bound to lysozyme (Lys+ThT) and insulin (Ins+ThT) amyloid fibrils states. CD spectra of (g) lysozyme and (h) insulin amyloid fibrils in the absence (Lys, Ins) and in the presence of fluorescent probes ANS (Lys+ANS, Ins+ANS) and ThT (Lys+ThT, Ins+ThT).

Mature amyloid fibrils were visualized using transmission electron microscopy (TEM) (Figure 1, left panels). It turned out that the amyloids formed from insulin and lysozyme, prepared under the selected conditions, are thin unbranched fibers with a tendency to form clots. It can be noted that the tendency to clustering of insulin amyloid fibrils is higher than that of lysozyme amyloid fibrils (fewer individual fibers that do not form fibrillar clots are observed). This fact is additionally confirmed by a higher value of turbidity and Rayleigh Light Scattering (RLS) of insulin amyloid fibrils (Figure 2(a-b)).

The circular dichroism (CD) spectra of both samples are specific for proteins with a predominantly beta-sheet structure (Figure 2(g-h)). At the same time, the CD spectra of amyloid fibrils formed from different proteins noticeably differ in shape and amplitude, which indicates the difference in the secondary structure of the insulin and lysozyme molecules forming amyloid fibrils.

### ANS may affect the secondary structure of mature amyloid fibrils and their clustering, while ThT does not have such an effect

The effect of ThT and ANS fluorescent probes on mature amyloid fibrils was analyzed with a wide range of physico-chemical approaches. It was shown that in the presence of ThT turbidity and RLS of the fibrils are practically unchanged (Figure 2(a-b)). A similar result was obtained in the presence of ANS in a sample with insulin amyloid fibrils. However, when ANS was injected into the sample with lysozyme amyloid fibrils, we observed a marked increase in turbidity and RLS, which may indicate increase in the number and/or physical dimensions of the studied aggregates. Electron microscopy data allowed us to suggest that the presence of ANS promotes additional clustering of lysozyme amyloid fibrils (Figure 1, top panels), not involved in the formation of clots in the stock sample.

In addition, it turned out that the presence of ANS in the samples leads to a significant change in the CD spectra of amyloid fibrils formed from the both proteins, and hence their secondary structure (Figure 2(g-h)). In particular, the minimum of the CD spectra of the samples, specific for the beta-folded structure, becomes less pronounced after the addition of ANS, which may indicate a decrease in the fraction of beta-sheets in the protein forming amyloid fibrils. At the same time, the presence of ThT had no effect on the CD spectra of the samples. A mechanism of the dyes interaction with fibrils was further analyzed.

### Fluorescent probes ThT and ANS have different amyloid fibril binding sites

The results of confocal fluorescence microscopy prove that ThT and ANS bound to amyloids (Figure 1, right panels). The photophysical characteristics of the dyes bound to fibrils were investigated using spectroscopic approaches. During ThT interaction with lysozyme amyloid fibrils, we observed a slight change in the shape of the dye absorption spectrum (the appearance of a shoulder in the long-wavelength region of the spectrum) (Figure 2(e)). When the dye binds to insulin amyloid fibrils, its absorption spectrum shifts noticeably to the long-wavelength region and changes its shape (Figure 2(e)), which is in good agreement with the results obtained by us earlier [20,45]. ThT fluorescence intensity increases more significantly when dye interacts with insulin amyloid fibrils than when it binds to lysozyme amyloid fibrils (Figure 2(f)). In the case of ANS, the opposite situation was observed: a more noticeable change in the shape of the absorption spectrum and the fluorescence intensity of the dye was observed when it interacts with lysozyme amyloid fibrils (Figure 2(c-d)). On the basis of these results, it was concluded that there is no reason to believe that ThT and ANS interact with the same binding sites of amyloids. Differences of photophysical characteristics of the dyes bound to fibrils allowed us to prove the difference in the probes binding but do not explain how they bind.

The mechanism of ThT interaction with amyloid fibrils was previously described by Krebs [46]. According to these ideas, the dye incorporates into the grooves formed by beta-sheets of fibrils along the long axis of the fibril fiber perpendicular to the beta-sheets. ANS binds to the exposed hydrophobic patches/surfaces of proteins, as well as integrate into hydrophobic cavities between protein associates forming large aggregates [36,38,47]. Since, according to our results, the interaction of ThT and ANS with fibrils is different, the ANS binding sites are apparently located outside the grooves along the long axis of the fibril, which could provide a hydrophobic microenvironment of the dye. Another variant for ANS interaction with hydrophobic regions of amyloid fibrils may be the integration of the dye into the area of their association. In order to verify this assumption, we ultrasonicated the amyloid fibrils to reduce the size of fibrillar clots.

### The binding of ANS to amyloid fibrils is probably largely due not to hydrophobic but to electrostatic interactions

**Figure 3.**
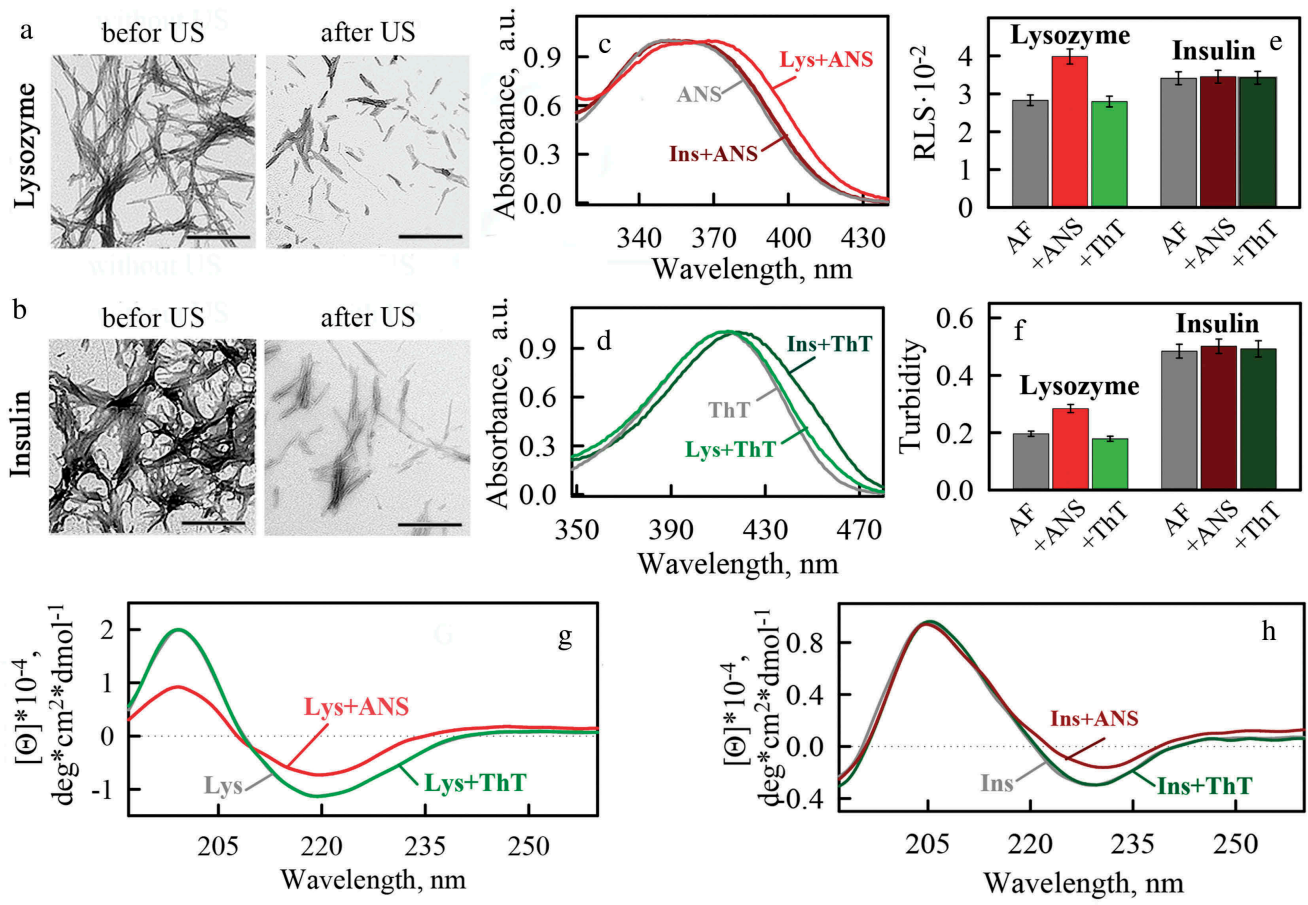
Morphology and photophysical properties of ultrasonicated amyloid fibrils formed from lysozyme and insulin using of buffer solution with pH 6.3 and and their binding to fluorescent probes ThT and ANS. (a), (b) – transmission electron microscopy (TEM) images of amyloid fibrils formed from lysozyme and insulin, respectively, before and after ultrasonication. The scale bars on the images are equal to 500 nm. (c) – normalized absorbance spectra of ANS in free (ANS) and bound to lysozyme (Lys+ANS) and insulin (Ins+ANS) amyloid fibrils states. (d) – normalized absorbance spectra of ThT in free (ThT) and bound to lysozyme (Lys+ThT) and insulin (Ins+ThT) amyloid fibrils states. (e) – Rayleigh Light Scattering (RLS) and (f) turbidity (at λ = 530 nm) of amyloids. CD spectra of (g) lysozyme and (h) insulin amyloid fibrils in the absence (Lys, Ins) and in the presence of fluorescent probes ANS (Lys+ANS, Ins+ANS) and ThT (Lys+ThT, Ins+ThT).

Ultrasonication of insulin and lysozyme amyloids, as expected, led to their defragmentation and a decrease in the size of fibrillar clots (Figure 3(a-b)). It is turned out that the absorption spectra of ANS (like ThT) remained practically unchanged, which indicates that ultrasonication did not affect the dye binding sites (Figure 3(c-d)). The effect of ANS to the samples turned out to be similar to that before fibrils ultrasonication: there was an increase in turbidity and RLS of the samples (Figure 3(e-f)), as well as a significant change in the shape of their CD spectrum (Figure 3(g-h)). The absence of any effect of ThT to the fibrils was also noted: turbidity, RLS, and CD spectra of the samples did not change (Figure 3(e-f)). Thus, despite the significant change in the morphology (reduction in the number and size of fibrillary clots) of amyloid fibrils formed from insulin and lysozyme under the influence of ultrasonication, the interaction of fluorescence probes with them practically did not change.

Obtained results indicate that ANS does not incorporate both into the grooves along the long axis of the fibril, and also into the regions of amyloid fibrils association. Thus, we were unable to identify hydrophobic areas/cavities in amyloid fibrils or their clusters for ANS binding.

The literature discusses that not only hydrophobic [48], but also electrostatic interactions [49] can underlie binding of ANS to proteins. In particular, it was suggested that ionic interaction between negatively charged ANS sulfonate groups with positively charged amino acids, for example, histidine, lysine or arginine, is the predominant type of interaction [50]. Perhaps this is the type of ANS binding that occurs in the case of amyloid fibrils.

### A change in the secondary structure and morphology of amyloid fibrils does not change the effect of ANS on amyloids

We tried to check whether a change in the secondary structure and morphology of amyloid fibrils (while the primary sequence of the amyloidogenic protein is unchanged) affects ANS binding and its effect on amyloids. To change the structure of aggregates formed from insulin and lysozyme, their fibrillogenesis was induced under altered conditions: proteins were dissolved in a buffer with extremely acidic pH (pH 2) in the presence of NaCl and incubated at a temperature of 37°C [31]. After the formation of mature amyloid fibrils, they were transferred to distilled water, and their stability was experimentally confirmed.

**Figure 4.**
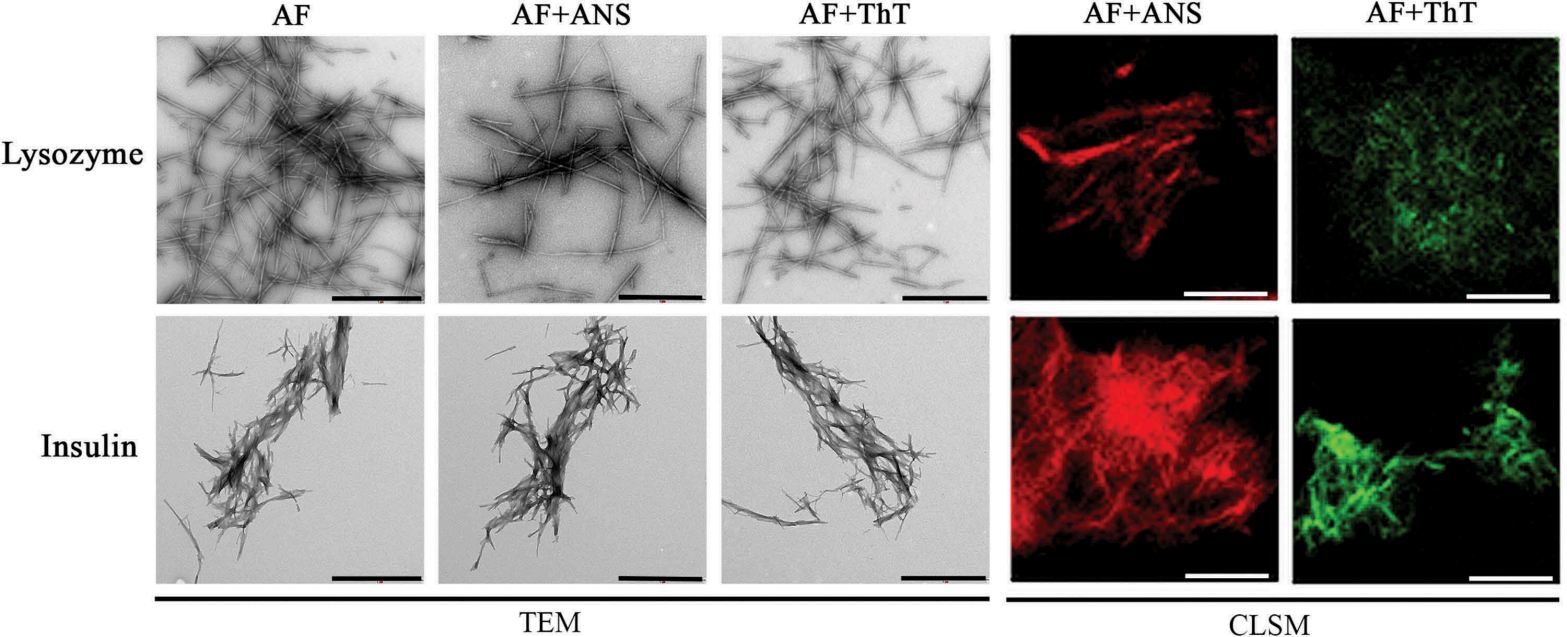
Morphology and staining by fluorescent probes of amyloid fibrils formed from lysozyme (top panels) and insulin (bottom panels) using of buffer solution with pH 2. Left panels show transmission electron microscopy (TEM) images of amyloid fibrils in the absence (AF) and in the presence of fluorescent probes ANS (AF+ANS) and ThT (AF+ThT). Data of confocal laser scanning microscopy images (CLSM, Right panels) show amyloids stained by these fluorescent probes. The scale bars on the TEM and CLSM images are equal to 1 and 4 µm, respectively.

**Figure 5.**
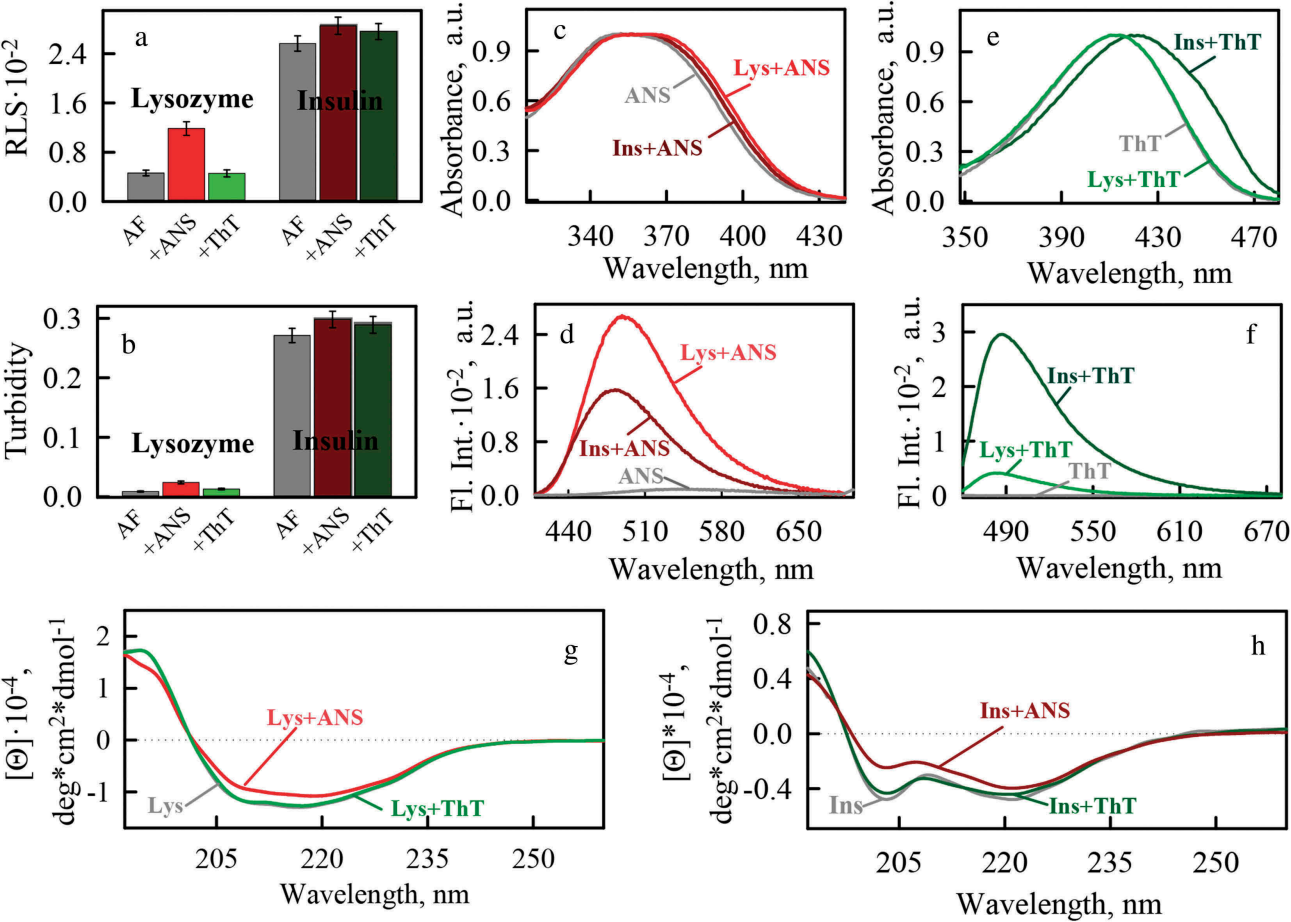
Photophysical properties of amyloid fibrils formed from lysozyme and insulin using of buffer solution with pH 2 and their binding to fluorescent probes ThT and ANS. (a) – Rayleigh Light Scattering (RLS) and (b) – turbidity (at λ = 530 nm) of amyloids. (c) – normalized absorbance and (d) – fluorescence spectra of ANS in free (ANS) and bound to lysozyme (Lys+ANS) and insulin (Ins+ANS) amyloid fibrils states. (e) – normalized absorbance and (f) fluorescence spectra of ThT in free (ThT) and bound to lysozyme (Lys+ThT) and insulin (Ins+ThT) amyloid fibrils states. CD spectra of (g) lysozyme and (h) insulin amyloid fibrils in the absence (Lys, Ins) and in the presence of fluorescent probes ANS (Lys+ANS, Ins+ANS) and ThT (Lys+ThT, Ins+ThT).

The insulin amyloid fibrils prepared under the chosen conditions are a thick bundle of intertwined fibrils (Figure 4, bottom left panels), while the amyloids obtained at more alkaline pH resembled a network of fibrillar fibers (Figure 1, bottom left panels). It turned out that lysozyme fibrils obtained at an acidic solution pH interact to one another a much lesser extent (Figure 4, top left panels) compared to fibrils prepared at a more alkaline solution pH (Figure 1, top left panels). The data on the lower degree of clustering, and therefore the smaller the size of the fibrillar clots, are in a good agreement with the very low values of turbidity and RLS of the sample (Figure 5(a-b)).

The CD spectra of both samples (Figure 5(g-h)) differ significantly from that of fibrils prepared on the basis of the same proteins at a more alkaline solution pH (Figure 2(g-h)). A change in the conditions of fibrillogenesis led to the appearance in the CD spectra of fibrils an additional short-wave shoulder along with the minimum specific for proteins with a predominantly beta-folded secondary structure. Thus, a change in the conditions of insulin and lysozyme fibrillogenesi led to a change in the secondary structure and morphology of their amyloid fibrils.

Using confocal fluorescence spectroscopy, it was shown how ThT and ANS stain insulin and lysozyme amyloids obtained under new conditions (Figure 4, right panels). Changes in the structure of amyloid fibrils led to a change in the shape of the absorption spectra and the fluorescence intensity of the dyes (Figure 5(c-f)). However, the fact that ThT binds more efficiently to insulin amyloid fibrils and ANS binds more efficiently to lysozyme amyloid fibrils remains unchanged. In addition, despite the low turbidity and RLS of lysozyme fibrils prepared at acidic pH of the solution, it was shown that the presence of ANS leads to a marked increase in these characteristics, while ThT does not have such an effect (Figure 5(a-b)).

In addition, it was shown that the presence of ANS in the samples leads to a significant change in the CD spectra of amyloid fibrils formed from both proteins, and hence their secondary structure. At the same time, the presence of ThT had no effect on the CD spectra of the samples. Thus, despite the change in the secondary structure and morphology of amyloid fibrils formed from insulin and lysozyme, the effect of ANS on them has not changed.

## Discussion

ThT and ANS fluorescent probes are widely used to study amyloid fibrils. According to the previously proposed model, ThT incorporates into the grooves formed by beta-sheets of fibrils along the long axis of fiber perpendicular to the beta-sheets [46]. This type of binding determines the high specificity of the interaction of the dye with the proteins in the state of amyloid fibrils, where the beta-folded structure is relatively rigid and strictly ordered (stacked). With this interaction, the intramolecular mobility of ThT molecule is limited, which leads to an increase in its fluorescence intensity [22]. Our results show that ThT binding to mature amyloid fibrils formed from insulin and lysozyme does not affect the object of the study. Experiments after changing of fibrillogenesis conditions (that led to change in amyloids structure) and their ultrasonication (with changing of their clasterization and morphology) led to the same results. Thus, we made a conclusion that ThT does not affect the structure and morphology of amyloid fibrils and can be used for their study without fear of incorrect results.

According to the literature ANS interacts with hydrophobic cavities of proteins (or their aggregates) [48]. This probe fluorescence is quenched by water [51] and significantly increases when this dye binds to hydrophobic areas of the proteins. Our results indicate that ANS does not integrate into grooves along the long axis of the fibril, which could provide a hydrophobic microenvironment for the dye molecule. In addition, it was shown that ANS does not incorporate into hydrophobic pockets in areas of amyloid clustering. Thus, we were unable to identify hydrophobic areas/cavities in amyloid fibrils or their clusters for ANS interaction. In this regard, we assume that the binding of the dye to amyloid fibrils is due not to hydrophobic, but to electrostatic interactions between the dye sulfonate group and amyloid-forming protein cationic groups. At the same time, we experimentally prove that ANS binding to all investigated types of amyloids accompanying by the significantly increase in the probe fluorescence intensity. The assumption that ANS binding does not require pre-existing hydrophobic sites in protein molecules to start dye-protein binding reaction is in a good agreement with early works [50,52]. Results of these works indicate that the protein does not behave as a static particle when ANS binds to it. Rather, ANS can induce protein conformational changes that lead to creation a hydrophobic microenvironment for the dye molecule and cause it to fluoresce. In particular, it is known that ANS and bis-ANS (dimeric analogue of ANS) can convert folded globular proteins into molten globule-like conformation (e.g. [53]). Alternatively, it was shown that unfolded proteins can be forced to fold into molten globule-like conformation by ANS [54]. In both cases, the observed structural changes were explained in terms of the ANS-induced shift of the conformational equilibrium towards the molten globule state leading to the high affinity of ANS.

In the present work we showed conformational changes of insulin and lysozyme molecules formed the amyloid fibril during their binding to ANS (Figures 2(g-h), 3(g-h) and 5(g-h)). Electrostatic interaction of ANS with amyloid fibrils should lead to decrease of the amyloid-forming molecules positive charge. Weaker electrostatic repulsion between positive charges of amyloids (at a certain ratio of negatively charged ANS to positively charged protein amino acid residues) enables fibrils interaction with each other. The described effects probably lead to the clustering of lysozyme fibrils in the presence of ANS.

It is important to note that ThT does not have such an effect to amyloid fibrils. This fact confirms the difference in the mechanisms of interaction of dyes with fibrils. The results of the work also indicate that with a change in the conditions of amyloid fibrils preparation and, consequently, a change in their structure, as well as with defragmentation of amyloids by ultrasonication, the influence of ANS on fibrils does not change, which prove the universality of the observed effects.

**In conclusion**, the results of the present work show that ANS should be used cautiously for the study of amyloid fibrils, since this fluorescence probe have a direct effect on the object of the study.

## Materials and methods

### Materials

Fluorescent dyes thioflavin T (ThT) ‘UltraPure Grade’ (AnaSpec, USA) and 8-anilino-1-naphthalene sulfonate (ANS) (Serva, Germany), lysozyme, insulin and buffer components (Sigma, USA) were used without after purification.

### Amyloid fibrils preparation and ultrasonication

For the preparation of amyloid fibrils proteins (2 mg/ml) were incubated with constant agitation (500 rpm) for 1 day in a TS-100 Thermo-Shaker (Biosan, Latvia) in two different conditions: in 100 мМ KH_2_PO_4_-NaOH in the presence of 3 М GdnHCl (pH 6.3) at 57°C and in 20% acetic acid solution in the presence of 100 mM NaCl (pH 2.0) at 37°C. After the formation of mature amyloid fibrils, they were transferred to deionized water by dialysis.

A water bath-type ultrasonic transmitter Elmasonic P30H with temperature controller (Elma GmbH, Germany) was used for decreasing of fibrillary clusters and defragmentation of amyloid fibrils. The volume of the water bath was about 2.5 L. The instrument frequency was 37 kHz, and the power output was set to deliver a maximum of 400 Watts. Samples were ultrasonicated from three directions (i.e., two sides and bottom) for 5 min at 37°C.

### Spectroscopic studies

The absorption spectra of the samples were recorded using a U-3900H spectrophotometer (Hitachi, Japan). The absorption spectra of amyloid fibrils and ThT in the presence of the fibrils were analyzed along with the light scattering using a standard procedure [55]. The concentrations of ThT and ANS and fibrils formed from lysozyme and insulin were determined using molar extinction coefficients of ϵ_412_ = 31600 M^−1^cm^−1^, ϵ_350_ = 5000 M^−1^cm^−1^, ϵ_280_ = 36000 M^−1^cm^−1^ and ϵ_280_ = 5800 M^−1^cm^−1^, respectively. Turbidity of the samples containing fibrils and fluorescent dyes was monitored by measuring absorbance at 530 nm.

Fluorescence spectra were measured using a Cary Eclipse spectrofluorimeter (Varian, Australia). For Rayleigh light scattering (RLS) determination samples with fibrils were excited at 530 nm and registered at 530 nm. Fluorescence of ThT was excited at a wavelength of 450 nm. Fluorescence of ANS was excited at a wavelength of 350 nm. The spectral slits width was 5 nm in most of experiments. Changing the slit widths did not influence the experimental results. Recorded fluorescence intensity was corrected on the primary inner filter effect with the use of previously elaborated approach [56].

Circular dichroism (CD) spectra in the far UV-region were measured using a J-810 spectropolarimeter (Jasco, Japan). Spectra were recorded in a 0.1 cm cell from 260 to 200 nm. For all spectra, an average of three scans was obtained. The CD spectrum of the appropriate buffer was recorded and subtracted from the samples spectra. Amyloid fibrils in concentration 0.2 mg/ml were used.

### Electron microscopy

Micrographs were obtained using a transmission electron microscope Libra 120 (Carl Zeiss, Germany). Amyloid fibrils were placed on cooper grids coated with formvar/carbon films (Electron Microscopy Sciences, USA). To obtain electron micrographs, the method of negative staining with a 1% aqueous solution of uranyl acetate was used.

### Confocal microscopy

For visualization of amyloids stained by fluorescent probes the confocal laser scanning microscope Olympus FV 3000 (Olympus, Japan) was used. The fixed excitation laser line (405 nm) was chosen (the choice is determined by the available lasers with discrete wavelengths closest to the absorption maxima of the dyes). Registration of fluorescent light was carried out in the range of 420–520 nm. An objective with a 60x magnification and numerical aperture NA 0.6 was used.
